# Surgical outcome of pediatric abdominal trauma at Tertiary Hospital, Northwest Ethiopia, a 3-year retrospective study

**DOI:** 10.1186/s12893-024-02493-1

**Published:** 2024-07-08

**Authors:** Yohannis Derbew Molla, Desyibelew Chanie Mekonnen, Andinet Desalegn Beza, Hirut Tesfahun Alemu, Deresse Abebe Gebrehana

**Affiliations:** 1 Department of Surgery, College of Medicine and Health Sciences, University of Gondar, Gondar, Ethiopia; 2 Department of Internal Medicine, College of Medicine and Health Sciences, University of Gondar, Gondar, Ethiopia; 3 College of Medicine and Health Sciences, University of Gondar, Gondar, Ethiopia

**Keywords:** Pediatric, Surgical, Outcome, Abdomen, Blunt, Penetrating

## Abstract

**Introduction:**

Abdominal trauma in children is a significant health concern that often leads to severe complications and even death. Children experience trauma more frequently than adults, with approximately one in four pediatric patients suffering from serious abdominal injuries. Falls are the leading cause of such trauma among children, which is intriguing considering that most hospital admissions for pediatric abdominal injuries result from blunt force impacts.

**Method:**

A retrospective cross-sectional analysis of medical records was conducted to examine all patients under the age of 16 who underwent exploratory laparotomy for abdominal trauma between January 1, 2020, and January 1, 2023. The clinical data were gathered using a data extraction sheet from each patient’s medical charts. Information on socio-demographic characteristics, injury mechanisms, clinical conditions at the initial presentation, intraoperative findings and complications, and patient outcomes were collected.

**Results:**

The study involved a total of 90 patients, consisting of 72 males (80%) and 18 females (20%). The average age of the patients was 10, with a standard deviation of 4.7, ranging from 2 to 16 years. Half of the patients were below the age of 10, accounting for 46 individuals (51%). The causes of the injuries varied among patients below and above the age of 10. However, overall, bullet injuries were the most common cause, accounting for 28 cases (31%), followed by falls in 21 cases (23.3%), horse or donkey kicks in 10 cases (11.1%), stab injuries in 10 cases (11.1%), horn injuries in 7 cases (7.8%), and road traffic accidents in 6 cases (6.7%). Physical assault, blasts, and other types of injuries, such as hen bites and metal rod injuries, were observed in 8 cases (8.9%) of the patients. Fall accidents, horse or donkey kicks, and horn injuries were particularly more common among children below the age of 10, while bullet injuries and stab injuries were most common among those above the age of 10.

**Conclusion:**

Following abdominal trauma in children, a range of complications may arise, including immediate issues such as infections and hemorrhaging, as well as long-term consequences like impaired organ functionality and psychological distress. In our region, young children are particularly vulnerable to accidents involving falls, kicks from horses or donkeys, and injuries caused by horns. Therefore, it is crucial to educate parents about these hazards. Additionally, providing appropriate guidance on firearm safety is essential for individuals who possess such weapons.

## Introduction

Pediatric abdominal trauma is a significant factor contributing to morbidity and mortality in children. Compared to adults, children experience trauma more frequently, and 25% of pediatric patients with serious trauma also have abdominal injuries. Falls are the most common cause of trauma in children, and blunt trauma is responsible for the majority of admissions for abdominal trauma [1, 2]. In the pediatric age range, trauma is the leading cause of death. Approximately 90% of cases of abdominal trauma in children are the result of blunt trauma, while 10% are the result of penetrating trauma [3]. The age and developmental stage of a child can influence the mechanisms behind abdominal trauma. Older children are more likely to sustain injuries from sports-related incidents and auto accidents, while younger children may suffer injuries from falls or non-accidental harm [4].

A comprehensive approach that incorporates laboratory testing, modern imaging techniques, and clinical assessment is necessary for diagnosing abdominal trauma in children. Computed tomography (CT) scans have become essential in the diagnostic process due to their ability to provide precise information about the severity of injuries. However, concerns about radiation exposure in young patients have prompted research into alternative imaging modalities such as magnetic resonance imaging (MRI) and ultrasound [5, 6]. CT tests always require bi-dimensional reconstructions on sagittal and coronal images, as they are crucial for determining the extent of the injury. Additionally, 3D reconstructions with volume rendering (VR) can be used to evaluate bone components [7].

In order to optimize outcomes, collaboration among pediatric surgeons, radiologists, and critical care specialists is crucial in the treatment of pediatric abdominal trauma. In certain cases, non-operative therapy has gained prominence, with a focus on regular physical examinations, vigilant monitoring, and imaging studies to guide decisions regarding surgical intervention. However, when significant organ damage, peritonitis, or hemodynamic instability is present, prompt surgical intervention is imperative [8, 9].

The aim of our study was to identify the etiological factors of abdominal trauma, the prevalence of intra-abdominal organ injuries, and surgical management and treatment outcomes in pediatric patients operated on for abdominal trauma at University of Gondar Comprehensive Specialized Hospital. As the sole referral hospital within the city, this hospital encompasses an Adult Trauma Unit with a pediatric team spearheaded by pediatric surgeons. Notably, the hospital lacks an Interventional Radiology department and a structured pre-hospital team. Most patients initially seek care at neighboring health centers before being referred to the hospital. Ambulance transport is typically utilized, accompanied by healthcare professionals.

## Methods and material

### Study design, setting and population

A retrospective cross-sectional analysis was conducted to analyze all cases of exploratory laparotomy for abdominal trauma in patients under the age of 16 between January 1, 2020, and January 1, 2023. The care of these patients involved senior general surgeons and senior general surgery residents who were closely supervised. The main source of data for this study was the patients’ medical records, which were identified using the operation theater register. Out of a total of 103 patients who underwent the procedure, only 90 records could be located due to some medical records being lost. This study was conducted in a city with a population of 323,900, as reported by the central statistical agency of the country in 2015 [10]. The city is home to one of the oldest teaching hospitals in the nation, which is a public comprehensive specialized facility. This hospital serves the healthcare needs of over 7 million residents in both the city and its surrounding areas [11]. After admission a comprehensive diagnostic protocol was employed. This protocol encompasses the immediate surgical intervention for individuals exhibiting clear indications. Additionally, it includes the utilization of classic emergency room examinations, such as FAST (Focused Assessment with Sonography in Trauma), pelvis and chest X-rays, and computed tomography (CT) scans, as deemed appropriate.

### Data collection

The clinical data were obtained utilizing a data extraction sheet from the medical charts of each patient. Information regarding socio-demographic characteristics, injury mechanisms, clinical conditions upon initial presentation, intraoperative findings and complications, as well as patient outcomes, were meticulously collected. Patients were monitored in the hospital for a minimum of three days after the operation and were advised to attend follow-up appointments at the Pediatric Outpatient Clinic (OPD). During these appointments, the patients’ feeding histories and nutritional statuses were evaluated, while their wounds were thoroughly examined for indications of infection and inadequate healing progress. Patients with a stoma were scheduled for readmission after three months, during which a stoma reversal procedure was performed.

### Ethical approval

“Ethical clearance for this research study was obtained from the Ethics Review Committee of the School of Medicine, College of Medicine and Health Science, under reference number SBMLS/745/2023.”

### Data processing and analysis

The data were assessed for inconsistencies, coding errors, completeness, clarity, and missing values prior to entry. EPI DATA (version 3.1) was used for data entry, and the data was subsequently exported to IBM SPSS Statistics 25 for further cleaning and analysis. Descriptive statistical analysis such as frequency, percentage, cross tabulation, mean, standard deviation, median, and Interquartile range (IQR) were performed.

## Results

The study involved a total of 90 patients, with 72 males (80%) and 18 females (20%). The average age of the patients was 10, with a standard deviation of 4.7, ranging from 2 to 16 years. All patients who had abdominal trauma and received operative intervention were included in the study. Among the patients, 46 (51%) were below the age of 10. The causes of the trauma varied between patients below and above the age of 10. Overall, the most common cause of injury was bullet injury, accounting for 28 cases (31%). This was followed by fall down accidents in 21 cases (23.3%), horse/donkey kick in 10 cases (11.1%), stab injury in 10 cases (11.1%), horn injury in 7 cases (7.8%), and road traffic accidents in 6 cases (6.7%). There were also cases of physical assault, blast injuries, and other injuries such as hen bites and metal rod injuries, which accounted for 8 cases (8.9%) (Tables 1 and 2).

Fall down accidents, horse/donkey kick, and horn injuries were particularly more common in children below the age of 10, while bullet injuries and stab injuries were more common in those above the age of 10 (Table 2). There was a significant association (p-value < 0.05) indicating that penetrating (gunshot and stab) injuries are less likely in children below age 10 compared to those aged 11–16 (odds ratio < 1). In addition, There is a significant association (p-value < 0.05) suggesting that falls are more likely in children below age 10 compared to those aged 11–16 (odds ratio > 1) and There is a significant association (p-value < 0.05) indicating that Horse/donkey kick is much more likely in children below age 10 compared to those aged 11–16 (odds ratio > 1). However, the rest of the mechanisms of injuries (Road traffic accident, Assault by stick/stone/kick, Horn, Other (hen bite metal rod)) do not show significant associations (p-value > 0.05) between the two age groups (Table 3).

Common signs and symptoms observed in the patients included those consistent with peritonitis in 76 cases (84.4%), abdominal distension in 35 cases (38.9%), omental evisceration in 14 cases (15.5%), bowel evisceration in 10 cases (11.1%), shock in 8 cases (8.9%), and other symptoms such as respiratory distress, vomiting, and per-rectal bleeding in 11 cases (12.2%) (Table 4).

Based on the site of trauma, a total of 26 patients (28.9%) reported injuries to the abdomen without specifying a specific area. This was particularly common among young children, individuals who fell down, and patients involved in road traffic accidents. In contrast, 25 patients (27.8%) sustained injuries in the left lower quadrant area, while 16 patients (17.8%) had injuries in the right lower quadrant. Additionally, 10 patients (11%) had injuries in the right upper quadrant, and 8 patients (8.9%) had injuries in the thoracoabdominal, back, or flank areas.(Table 5). In terms of associated injuries, 8 patients (8.9%) experienced pneumothorax, while 7 patients (7.8%) sustained an extremity fracture. The median duration of presentation, from the time of injury to seeking medical attention, was 16.4 h. The range varied from 30 min to 96 h, with an interquartile range of 8 to 20 h.

All patients underwent surgery performed by experienced senior surgeons and senior general surgery residents to assess and manage their abdominal findings. Prior to surgery, patients were stabilized through preoperative measures such as resuscitation with normal saline or blood if necessary, insertion of a nasogastric tube, catheterization, and administration of antibiotics. During surgery, small bowel injury was observed in 58.9% of patients, while 26 patients (28.9%) had large bowel injuries. Other injuries included the spleen (18 patients, 20%), stomach (12 patients, 13.3%), liver (10 patients, 11.1%), and diaphragm (7 patients, 7.8%)(Table 6). Surgical procedures varied and included exploratory laparotomy and repair, resection and anastomosis, creation of a stoma, splenectomy, debridement, and other relevant procedures (Table 7).

After surgery, the majority of patients (88, 97.8%) were discharged with excellent recovery. However, 2 patients (2.2%) died due to a penetrating abdominal injury with shock upon admission and multiple organ injuries during surgery.

In terms of complications, 21 patients (23.3%) developed postoperative complications. These included pneumonia (5 patients, 5.6%), surgical site infection (3 patients, 3.3%), malnutrition (2 patients, 2.2%), necrotizing fasciitis (2 patients, 2.2%), severe hypokalemia (2 patients, 2.2%), and other complications (7 patients, 7.8%), such as empyema and wound dehiscence (Table 8). Two patients were readmitted for postoperative adhesion and managed conservatively.

**Table 1:** Sociodemographic characteristics of patients operated for abdominal trauma at hospital (*n* = 90)

Sociodemographic characteristics		Frequency (*n* = 90)	Percentage
Age	≤10	46	51.1
	11–16	44	49.9
Sex	Male	72	80
	Female	18	20
Residence	Urban	38	42.2
	Rural	52	57.8
Mechanism of injury	Gunshot	28	31.1
	Fall down	21	23.3
	Horse/donkey kick	10	11.1
	Stab	10	11.1
	Horn	7	7.8
	RTA	6	6.7
	Assault by stick/stone/kick	3	3.3
	Blast	2	2.2
	Others (Hen bite, metal rod)	3	3.3

**Table 2:** Mechanism of injury in those children less than 10 and greater than 10 years (*n* = 90)

Mechanism of injury	Below age of 10	Age between 11–16	Total
Gunshot	10	18	28
Fall down	15	6	21
Road traffic accident	4	2	6
Horse/donkey kick	9	1	10
Assault by stick/stone/kick	1	2	3
Horn	4	3	7
Stab	0	10	10
Blast	1	1	2
Other (hen bite, metal rod)	2	1	3

**Table 3:** The results of the Fisher’s exact test, along with the odds ratios and confidence intervals for each mechanism of injury in those children less than 10 and greater than 10 years (*n* = 90)

Mechanism of injury	AOR	*p*-value	CI Lower	CI Upper
Penetrating (gunshot and stab)	0.163	0.000108	0.065	0.408
Fall down	3.065	0.046030	1.063	8.836
Road traffic accident	2.000	0.677097	0.347	11.514
Horse/donkey kick	10.459	0.015321	1.265	86.463
Assault by stick/stone/kick	0.467	0.612360	0.041	5.338
Horn	1.302	1.000000	0.274	6.179
Other (hen bite metal rod)	1.955	1.000000	0.171	22.357

**Table 4:** Clinical presentation of patients operated for abdominal trauma (*n* = 90)

Clinical presentation	Frequency (*n* = 90)	Percentage
Peritonitis	76	84.4
Bowel evisceration	10	11.1
Omental evisceration	14	15.5
shock	8	8.8
Abdominal distension	35	38.9
Other signs or symptoms (vomiting, bleeding per rectum, fascial defect)	11	12.2

**Table 5:** Anatomic site of the trauma in the abdomen in patients operated for abdominal trauma (*n* = 90)

Anatomic site	Frequency (*n* = 90)	Percentage
Abdomen (non-specific region)	26	28.9
Left lower quadrant	25	27.8
Right lower quadrant	16	17.8
Left upper quadrant	10	11.1
Right upper quadrant	6	6.7
Thoracoabdominal	5	6.7
Back and flank	3	3.3

**Table 6:** Injured organs among patients operated for abdominal (*n* = 90)

Injured organs		Frequency (*n* = 90)	Percentage
Small bowel	Jejunum	29	32.2
	Ileum	22	24.4
	Duodenum	2	2.1
Colon	Transverse colon	14	15.5
	Sigmoid	5	5.5
	Other ( cecum, ascending, descending and rectum)	7	7.8
Liver		10	11.1
Diaphragm		7	7.8
Stomach		12	13.3
Spleen		18	20
Kidney		1	1.1
Pancreas		2	2.1
Bladder		4	4.4
Gallbladder		3	3.3
Others (retroperitoneal contusion/hematoma, wall defect)		5	5.6
Negative		6	6.7

**Table 7:** Performed procedures done in patients with abdominal trauma (*n* = 90)

Procedures performed	Frequency (*n* = 90)	Percentage
Repair (small bowel, colon, and solid organs)	62	68.9
Resection and anastomosis	19	21.1
Colostomy	14	15.6
Ileostomy	6	6.7
Splenectomy	4	4.4
Cholecystectomy	3	3.3
Damage control	1	1.1
Peritoneal lavage	3	3.3
Other (debridement, fascia reconstruction, foreign body removal)	14	15.6

**Table 8:** Complications seen among patients operated for abdominal trauma (*n* = 90)

Complications	Frequency (*n* = 90)	Percentage
Pneumonia	5	5.6
SSI	3	3.3
Electrolyte disturbances	2	2.2
Malnutrition	2	2.2
Necrotizing fasciitis	2	2.2
Others (empyema, dehiscence, UTI, Acute fulminant hepatitis)	7	7.8
Death	2	2.2

## Discussion

One of the primary factors contributing to pediatric morbidity and mortality is trauma. Globally, injuries and violent crimes are the leading causes of death among children. Nearly 90% of these cases involve unintentional injury. For individuals aged 10 to 19, trauma is the leading cause of death. Low- and middle-income countries account for approximately 95% of pediatric accident-related deaths. Among pediatric patients with significant trauma, around 25% experience abdominal trauma, which is the most common cause of undetected fatal injuries in children. Blunt trauma is the most prevalent form of pediatric abdominal trauma, and most children with this type of injury recover well without requiring surgery. Furthermore, patients who receive selective non-operative care for penetrating pediatric abdominal trauma achieve positive outcomes [1, 12].

A penetrating injury is characterized by the mechanical penetration of the skin and tissues by a foreign instrument, such as a knife, pistol, or other sharp object [13]. In our hospital, the most common types of penetrating trauma are gunshot and stab injuries. The severity of the damage caused by a penetrating injury is determined by the kinetic energy of the object that enters the body. When a knife applies low pressure to a small area of the body, it can still cause tissue damage. However, this low energy pressure typically does not affect the tissues surrounding the original site of injury. On the other hand, gunshot wounds have a high-pressure point that can cause serious damage to the surrounding tissues [14]. In Ethiopia, as well as in other parts of the world, there is a concerning trend of minors carrying firearms and using stabbing objects. The increasing number of incidents involving penetrating trauma may be attributed to factors such as lower socioeconomic class, growing wealth disparity, lack of knowledge, media amplification, improper gun handling, and increased accessibility to these dangerous items [15, 16].

There are numerous contributing factors that drive the prevalence of minors carrying firearms or knives in Ethiopia. One underlying reason is the widespread availability of weapons due to the country’s history of armed conflict and porous borders. Moreover, the absence of strict gun control laws and regulations exacerbates the issue. Another contributing factor lies in cultural norms and practices. In certain regions, carrying weapons is perceived as a symbol of masculinity, protection, and social status, particularly among young males. Additionally, poverty and lack of opportunities may lead some minors to engage in criminal activities, using weapons as a means of self-defense or intimidation. The consequences of minors carrying weapons are severe, ranging from accidental shootings and injuries to violent crimes. It is imperative to address these underlying factors and implement comprehensive measures, such as stricter gun control laws, community-based violence prevention programs, and educational campaigns, to reduce the prevalence of minors carrying weapons in Ethiopia.

Evaluating children who have experienced acute abdominal trauma can be challenging due to various factors. These include age-related physiological and developmental issues, changes in mental state, and the discomfort that adult caregivers may feel when caring for young patients. The current gold standard for diagnosing intra-abdominal injury (IAI) in both adults and children is computed tomography (CT) combined with cross-sectional imaging [17, 18]. When it comes to trauma, penetrating injuries typically affect the gastrointestinal (GI) system, specifically the jejunum, ileum, duodenum, colon, and stomach. On the other hand, blunt trauma primarily affects the spleen and kidney [19]. Our study found similar patterns of injury distribution.

Trauma surgeons have gained significant experience in managing spleen injuries without resorting to surgery. This organ has been extensively studied, and the lessons learned from treating spleen injuries have been applied to other solid abdominal organs. Despite their similarities, the spleen, liver, kidney, and pancreas have distinct morphologies and physiologies. While most solid organ injuries can be treated without surgery, the consequences may vary depending on the specific organ involved. One study data suggests that children with pancreatic injuries are more likely to require a delayed operation, while those with liver injuries are less likely to require surgical intervention after non-operative care. The liver is the second most commonly damaged organ in children who experience forceful abdominal trauma [20].

It is uncommon for blunt abdominal injuries to result in renal damage. However, due to anatomical differences, children are more susceptible to renal trauma due to less perirenal fat and weaker muscles. The majority of renal injuries are classified as grades I and II, indicating minimal damage. As a result, the non-operative treatment of blunt renal injuries has shifted, as it was discovered that more cases of nephrectomy were a result of surgical intervention [5, 6]. Similarly, pancreatic damage is also relatively rare in children. Historically, adults with pancreatic injuries were treated non-operatively. However, there has been a recent shift in the treatment of pediatric pancreatic injuries, with surgeons now utilizing non-operative measures [20, 21]. In our study only one and two patients had renal and pancreatic injuries respectively.

When evaluating abdominal and pelvic injuries in hemodynamically stable children following blunt trauma, computed tomography (CT) is the preferred imaging method. CT images are collected from the lower chest to the pubic symphysis. To minimize radiation exposure, it is recommended to avoid the basal phase in young children who have experienced trauma, after the scout view. Instead, an unenhanced CT scan can be performed to highlight the presence of free air, hematoma, and bone fractures in the abdomen. The use of oral contrast media is not advised due to the minimal benefits compared to the challenges it presents in individuals with severe injuries. Bidimensional reconstructions using sagittal and coronal images are essential for understanding the extent of the damage and should always be included in a CT examination. Additionally, 3D reconstructions with volume rendering can be utilized to evaluate bone components [7, 22, 23].

Patients who may benefit from a CT scan as part of their initial trauma evaluation can be identified using screening laboratory and imaging tests, along with other clinical characteristics. This approach takes into consideration the cost and potential long-term radiation risks associated with CT scans. However, within the pediatric trauma community, there is disagreement regarding the appropriate use of CT scans [24, 25]. Contrast-enhanced ultrasound (CEUS) has shown a sensitivity range of 85.7–100% and a specificity range of 89–100%. It appears to be a reliable and safe method for diagnosing abdominal solid organ damage in children with blunt abdominal trauma [26].

In recent years, there has been a significant shift in the non-operative management of abdominal injuries in children. The use of available resources has improved the diagnosis of pediatric abdominal injuries. Treatment decisions are now based on the patient’s hemodynamic state rather than the severity of the injury. Stable patients are discharged earlier, can return to school after their hospital stay, and are allowed to have lower hemoglobin levels before receiving a blood transfusion. Intensive care units (ICUs) are reserved for patients who have recently or continuously experienced bleeding, those who were previously unstable, or children with multiple traumas requiring ICU care. There is growing evidence supporting additional therapies and identifying risk factors for non-operative treatment failure. The available information is now sufficient to significantly change the management of abdominal injuries in children, and this knowledge is being incorporated into new evidence-based care algorithms [27].

Laparoscopy has emerged as a preferred treatment option for pediatric abdominal trauma, particularly in female patients who are younger and more stable after experiencing forceful injuries. This minimally invasive procedure has shown comparable or even superior results to laparotomy, with fewer unfavorable events and missed injuries. The growing use of laparoscopy in treating pediatric abdominal injuries reflects the increasing acceptance of this strategy [28]. In resource limited areas like ours, laparotomy is still the only option.

### Key findings of the research

- The research conducted was a retrospective study involving 90 pediatric patients who underwent exploratory laparotomy for abdominal trauma at a hospital in Ethiopia between 2020 and 2023.
- Among the children with abdominal trauma, the most common indications for laparotomy were gunshot wounds (31%), falls (23.3%), and kicks or stabs by animals or humans (22.2%).
- The small bowel was the most frequently injured organ (58.9%), followed by the colon (28.9%) and the spleen (20%).
- Regarding surgical procedures, repair of organ injuries was the most common (68.9%), followed by resection and anastomosis (21.1%), and creation of a stoma (22.2%).
- The overall mortality rate observed in the study was 2.2%. Postoperative complications included pneumonia (5.6%), surgical site infection (3.3%), and malnutrition (2.2%).

### Limitations of the study

- **Retrospective design**: The study relied on medical records, which may have incomplete, inaccurate, or missing data.
- **Small sample size**: The study involved only 90 patients, which may limit the generalizability and statistical power of the findings.
- **Lack of follow-up**: The study did not report the long-term outcomes of the patients, such as functional recovery, quality of life, or psychological distress.

### Recommendations

- We strongly advocate for the implementation of educational interventions aimed at increasing awareness and preventing common causes of pediatric abdominal trauma, such as falls, animal kicks, and horn injuries.
- It is imperative to enhance public awareness regarding firearm use and promote measures to prevent accidents.
- It is crucial to develop evidence-based guidelines and algorithms for the management of pediatric abdominal trauma, incorporating the latest advancements in imaging technology, a multidisciplinary approach, and non-operative therapy.
- We propose a future multicenter prospective study that compares the outcomes of operative and non-operative management for various types of abdominal organ injuries, including pancreatic, liver, and renal injuries. This study should also aim to identify the optimal criteria for selecting the most appropriate treatment modality.

## Conclusion

The clinical situation of pediatric abdominal trauma is complex and challenging, necessitating a profound understanding of the mechanisms of injury, clinical manifestations, diagnostic modalities, and treatment options. Advancements in imaging technology, in conjunction with a multidisciplinary approach, have facilitated the identification and management of these injuries. Abdominal trauma represents a significant surgical indication and contributes to morbidity and mortality rates in our region. Consequently, it is imperative to comprehend this grave condition. Additional research is required to enhance the comprehensive care provided to children with abdominal injuries, reduce radiation exposure, and optimize diagnostic algorithms.

## Data Availability

All data generated or analysed during this study are included in this published article.

## Abbreviations

BAT: Blunt abdominal trauma
CEUS: Contrast enhanced ultrasound
CT: Computed tomography
GI: Gastrointestinal
IAI: Intra-abdominal injury
ICU: Intensive care unit
IQR: Interquartile range
MRI: Magnetic resonance imaging
